# Using State Variables to Model the Response of Tumour Cells to Radiation and Heat: A Novel Multi-Hit-Repair Approach

**DOI:** 10.1155/2013/587543

**Published:** 2013-12-16

**Authors:** Stephan Scheidegger, Hans U. Fuchs, Kathrin Zaugg, Stephan Bodis, Rudolf M. Füchslin

**Affiliations:** ^1^ZHAW School of Engineering, Zurich University of Applied Science, 8401 Winterthur, Switzerland; ^2^University Hospital Bern, Switzerland; ^3^Radio-Onkologie-Zentrum KSA-KSB, 5001 Aarau, Switzerland; ^4^Medical Faculty, University of Zurich, 8006 Zurich, Switzerland; ^5^European Centre of Living Technology, 30124 Venice, Italy

## Abstract

In order to overcome the limitations of the linear-quadratic model and include synergistic effects of heat and radiation, a novel radiobiological model is proposed. The model is based on a chain of cell populations which are characterized by the number of radiation induced damages (hits). Cells can shift downward along the chain by collecting hits and upward by a repair process. The repair process is governed by a repair probability which depends upon state variables used for a simplistic description of the impact of heat and radiation upon repair proteins. Based on the parameters used, populations up to 4-5 hits are relevant for the calculation of the survival. The model describes intuitively the mathematical behaviour of apoptotic and nonapoptotic cell death. Linear-quadratic-linear behaviour of the logarithmic cell survival, fractionation, and (with one exception) the dose rate dependencies are described correctly. The model covers the time gap dependence of the synergistic cell killing due to combined application of heat and radiation, but further validation of the proposed approach based on experimental data is needed. However, the model offers a work bench for testing different biological concepts of damage induction, repair, and statistical approaches for calculating the variables of state.

## 1. Introduction

In radiation oncology, mathematical models are used to describe clonogenic survival, tumour control probabilities (TCP), or normal tissue complication probabilities (NTCP). The most widely used model for cell survival is the linear-quadratic (LQ) model. The (originally empiric) model was first used by Lea and Catcheside [1] to fit radiation chromosome damage. The model is based on the observation that the logarithmic plot of the surviving cell fraction *S* = *N*/*N*_0_ (with *N* = number of viable cells after and *N*_0_ number of cells before radiation) versus radiation dose *D* can be described by a linear and a quadratic dose-dependent term (log⁡ *S*(*D*) = −(*αD* + *βD* ^2^)). Based on this relationship, adaption of doses for hyper- or hypofractionated radiotherapies can be calculated (e.g., application of the BED concept in clinical oncology [2]). There is also a certain need to calculate equivalent doses in the case of application of moderate hyperthermia (40–43°C) in combination with radiation (HT-RT). But the extension to combined therapies requires some knowledge of the underlying dynamic processes (radiation and heat induced formation of cellular damages, repair, etc.).

Theories about DNA lesion formation or cell survival (e.g., Chadwick and Leenhouts [3]) led to mechanistic interpretations of the LQ model. Such interpretations are problematic, due to different problems related to the LQ-formulation. Criticism of the LQ model can be based on the following points.

1. For high doses, the shape of the survival plots is not linear-quadratic but shows a linear-quadratic-linear (LQL) behaviour [4].
2. Cell survival not only depends upon the radiation dose applied to the cells but also upon the dose rate [5]. Dose rate dependencies can in principle be included by a dose rate factor *q* (log⁡⁡*S*(*D*) = −(*αD* + *q* *βD* ^2^)) [1, 6], but the explicit calculation of this factor is limited to certain cases of repair kinetics (e.g., first- and second-order kinetics).
3. When applying well separated fractions of radiation (time gap larger than 24–48 h), the effect of previously applied radiation dose on the quadratic term in the LQ law (*βD* ^2^) fades away. In this context, Oliver [7] introduced a concept using a biological dose equivalent without taking a step toward a dynamic description of the system. Here as well, a dose rate factor or dose protraction factor can be introduced to correctly calculate the survival for split dose experiments or fractionated radiotherapy (with the same limitations as in point 2, if the time gap between the fraction is too short to ensure complete repair).
4. Survival curves are different for the different phases of the cell cycle. Mitotic cell response to radiation can be characterized in a logarithmic plot of the surviving cell fraction by a linear curve with a steep slope compared to the nonmitotic cells. A similar behaviour is proposed for apoptotic cells [8], whereas the nonapoptotic counterpart can be well characterized by a linear-quadratic curve using different radiation sensitivity coefficients. Also high LET-radiation is leading to a linear curve in the logarithmic survival plot, which has been interpreted in the framework of dual radiation action [9]. For all these reasons, the radiation sensitivity coefficients have to be adapted.
5. Some cell lines exhibit a phenomenon called low dose hypersensitivity [10, 11]. This seems to be a strong indication for a repair mechanism, which is triggered by the radiation. The resulting logarithmic survival curve is far from a simple linear-quadratic shape.
6. If radiation is applied in combination with moderate hyperthermia (typical temperatures between 40°C and 43°C), a synergistic effect between radiation and heat can be observed [12]. Heat seems to act as radiosensitizer but does not kill cells directly below 44°C. In principle, the radiosensitivity coefficients of the LQ model can be regarded as temperature dependent, but the effect is also depending upon the duration of heating and the time gap between heating and irradiation. A set of coefficients is therefore only valid for a well-defined application of heat and radiation. Interestingly, both heat prior and after irradiation have an impact on cell killing [13]. This seems to be a result of the underlying dynamic processes, which are not covered by adapted LQ models.

The points listed here (1–6) have a common aspect. Linear-quadratic-linear shapes, dose rate dependence, repair during fractionated radiotherapy, different shapes of survival curves for different situations, and so forth are based on dynamic processes. The use of the LQ-formula and the adaption of the radiosensitivity parameters to a specific situation [14] may be used at best for describing experimental data but definitely does not contribute to a profound understanding of the biological system. Therefore, the mechanistic interpretation of the LQ model seems to be problematic.

It is important to point out the intension behind modelling. The aim of modelling can be prediction,—for example, in the case of radiation oncology the prediction of TCP for a modified fractionation. In our view at least similarly important goal is the effect upon learning. Dynamic modelling can be used to test ideas about the dynamics in a system. Modelling requires making ideas and concepts explicit. This leads to revisions of the ideas often before the results of model based computer simulation are available. The proposed model framework in this paper will focus on modelling as basis for in silico experiments helping us to learn about the relevant dynamic processes responsible for cellular response to radiation only or radiation and heat. Prediction is then considered a subsequent goal.

To overcome the limitations of the LQ model, dynamic models using ordinary differential equations (ODE) have been developed. A good example is the lethal-potentially-lethal- (LPL) model of Curtis [15]. The model describes the formation of lethal and potentially lethal DNA lesions and is able to describe the linear-quadratic-linear behaviour of the logarithmic survival curves and to fit the dose rate dependencies observed by Wells and Bedford [5]. The surviving fraction is calculated by using Poisson statistics. This makes the inclusion of or extension to the dynamic interplay between different tumour subpopulations hard. Non-Poisson approaches have been evaluated by Vassiliev [16]. The proposed multi-hit model is not a dynamic model based on ODE. In contrast to a model based on the calculation of DNA lesions, a population based model offers a more natural and direct approach to the dynamic aspects at the tissue level (interaction of tumour cells with host tissue and vascularisation or forming of subpopulations with a different radiosensitivity as observed *in vivo*). The extension of the model to intratumour heterogeneity seems to be highly important since malignancy of tumours is coupled with genetic instability [17]. This was the main motivation for developing the Γ-LQ model [18]. The key idea of this model is the use of a differential equation for cell killing (for which the LQ law is a solution) and to substitute the absorbed dose *D* by a biological dose equivalent Γ. This biological dose equivalent is assumed to be proportional to the radiation damage relevant to cell death. Cellular repair is considered by a kinetic model for this dose equivalent. With this model, it is also possible to reproduce the linear-quadratic-linear behaviour of large fraction doses and to approach dose rate dependence similar to the LPL model of Curtis. The Γ-LQ model has been extended to the synergistic effect of heat and radiation [19].

**Figure 1:**
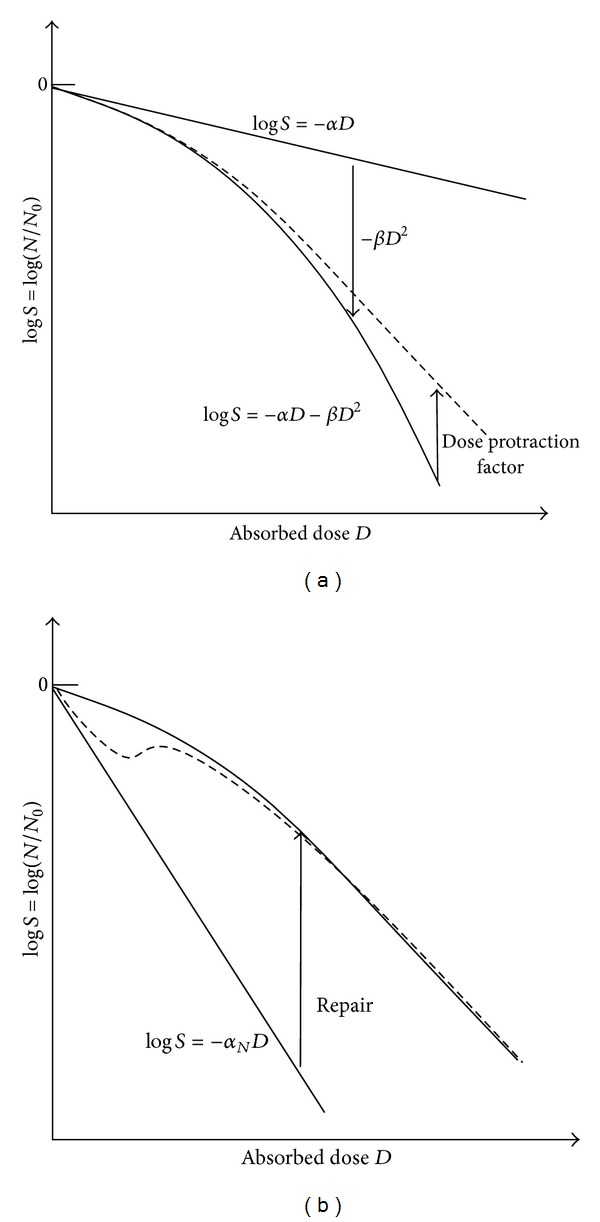
Comparison of two different concepts for describing cell killing or cell survival (schematic illustration). The left diagram (a) shows a correcting down approach (bending down principle by a term describing additional cell killing due to previously acquired sub-lethal lesions). To correct the curve to the observed linear-quadratic-linear shape and to include dose rate dependences, dose protraction factors (e.g., Lea-Catcheside dose protraction factor [1, 6]) have been proposed. The right diagram (b) illustrates a correcting up approach due to the repair of potentially lethal lesions. If the activation of repair will need a certain dose, low dose hypersensitivity (dashed curve) can be explained [23].

All LQ-type models including dynamic models such as the Γ-LQ model and also models for DNA lesions kinetics (e.g., model of Curtis) are based on the following biological concept. Lethal damages are not reparable and are produced depending linearly upon the dose rate *R* = *dN*/*dt* (e.g., LQ model with number of viable cells *N* : *dN*/*dt* = −*αR*). This leads to a linear graph in the logarithmic survival plot (∫*dN* = −*α*∫*R* *dt* = −*αD*). An additional cell killing occurs due to sublethal DNA lesions produced by previously applied radiation doses. In the LQ-type model, this is realised by a second term (−*βD* ^2^). In principle, the LQ law can be interpreted as a solution of the following ODE [18]: *dN*/*dt* = (−*α* − 2*βD*) · *NR*. The cell killing part of the Γ-LQ model is derived by substituting the absorbed dose *D* by the biological dose equivalent Γ : *dN*/*dt* = (−*α* − 2*β*Γ) · *NR*. In each of these formulations, the linear graph in the logarithmic survival plot is bent downward by an additional term to a linear-quadratic or a linear-quadratic-linear shape (Figure 1). In this view, the linear-quadratic behaviour is a result of a previous, remaining (not repaired) radiation damage. This damage can be considered as sublethal lesions which combined with lethal lesions by further radiation.

Observations of low dose hypersensitivity or the synergistic effect of moderate hyperthermia with radiation or even the fact that mitotic or apoptotic cells are exhibiting a steep slope of the log⁡⁡*S*-graph are indicating another type of underlying dynamic process. The linear-quadratic shape in the survival diagrams could be regarded as a result of the combination of induction of potentially lethal DNA damages and a repair process which is bending the log⁡⁡*S*-curve upward (Figure 1). The biological rationale for this type of dynamics is the following. Ionizing radiation produces DNA lesions with different degree of severity. It seems to be difficult to distinguish between lethal and nonlethal damages since double strand breaks can be repaired as well by homologue recombination or by non-homologues end-joining repair (NHEJ). A radiation dose of 1 Gy produces ca. 25 double strand breaks [20]. Depending on the cell line, cell surviving fraction is of the order of 70–80% [20]. Consequently, lethality would be a result of not repaired, possibly multiple double strand breaks. As soon as a repair process is activated, potentially lethal damages can fade away and the survival will increase.

Regarding the processes of repair and the role of repair proteins, cell survival or cell killing can be considered as a result of the dynamic interplay between DNA and (attached or associated) proteins. Possibly, this does not only include proteins of specific repair pathways but also proteins stabilizing the DNA strands (such as histones) and membrane proteins. In irradiated cells, DNA and proteins are exposed to radiation. In a mammalian cell nucleus, the number of ionisations is of the order of 10^5^ per 1 Gy of X-rays, most of them are ionisation of water molecules [20, 21]. At higher doses, severe damages of proteins may be expected as well due to ionisation and subsequent molecular changes leading to protein denaturation. This could result in a decreased repair capacity at higher doses and, in consequence, in a reduced upward correcting of the survival curve as illustrated in Figure 1. Such an approach may be supported by the observation that moderate hyperthermia (40–45°) is affecting proteins [12, 22]. These thermal damages seem to be responsible for the synergistic effect of heat combined with radiation.

A question regarding the dynamic interplay between (repair) proteins and DNA arises: can such a type of process explain the linear-quadratic or linear-quadratic-linear dose response of the survival. This leads to the following hypothesis: a model reflecting dynamic interaction of DNA (DNA damages triggering repair process) and proteins (responsible for repair and also susceptible to radiation damages) can describe aspects of the impact of radiation such linear-quadratic (-linear) behaviour and dose rate dependence of survival.

A key problem of such an approach is the handling of the complexity of biological systems. However, some aspects of biological systems may help to reduce the complexity of the model. First, biological systems are evolved—the biological control processes have certain robustness. As long as the cellular system is not driven too much away from its normal conditions, the cellular response may be covered by a low dimensional description. Second, the response of biological systems can be understood as an emergent phenomenon;—therefore, it could be helpful to use a phenomenological description of the observed dynamics instead of a molecular, mechanistic one. This idea is also supported by the fact that the evolutionary process is governed by the selection of the phenotype (semantic level) although the mutations occur on the molecular (syntactic) level. Some ideas inspired by statistical mechanics could be applied. Cellular damages on the molecular level may be approached by state variables. Similar to thermodynamic quantities such as entropy, variables describing cellular disorder (state of an ensemble) could be employed. Following the idea of a check sum principle, cellular response can be regarded as being governed by such state variables.

The Multi-Hit-Repair (MHR) model presented in this paper is based on a model framework that incorporates the idea of using variables of state. This framework allows expansion to synergistic interplay of heat and radiation. Therefore, the effect of heat and radiation is included. In the following sections, the model framework and MHR model will be clarified. The results using the MHR model as basis for computer simulations (in silico experiments) will focus on the radiation part. The model framework presented here is intended to offer a work bench for testing different ideas or hypothesis about cellular repair processes.

## 2. Materials and Methods

The first subsection concerning the general model (Section 2.1) describes a model framework which is referring to the relation between the different quantities/variables of state. Based on this, the state variables used in the MHR model will be defined (Section 2.2) and finally the influence of the variables of state upon cell killing will be modelled by a population model for tumour cells.

### 2.1. General Model Structure

**Figure 2:**
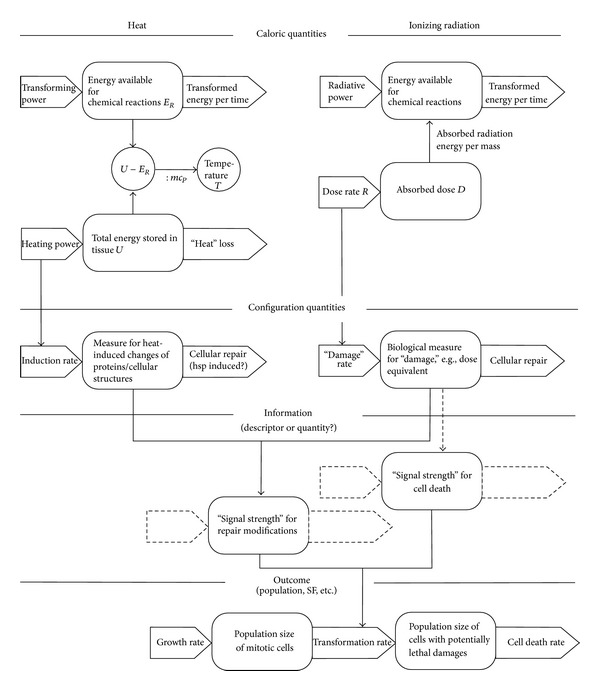
General model structure as a model framework. The structures consisting of boxes and thick arrows symbolise integrators. In the case of extensive quantities, the boxes can be regarded as storage elements and the arrows as flows. The left side of the diagram illustrates the effect of heat, the right side the effect of radiation. The population model is drawn in a simplistic manner. For the MHR model, a chain of population is used (see Section 2.3). In constructing this scheme, we have been critical of attempts to conflate concepts of thermodynamic entropy, statistical entropy, and information. We believe that distinguishing between the three (as caloric, number of configurations and information) leads to advances in understanding systems and processes. See also Corning and Kline [25, 26].

The model framework consists of different levels characterising different aspects of the biophysical system (Figure 2). At the top level in Figure 2, the physical quantities (caloric quantities) are illustrated. In the case of heat, more or less all molecules absorb energy (large amount of energy absorbed in a distributed manner). In contrast to heat, the energy absorption in the case of ionizing radiation occurs very locally. Szasz and Vincze [24] pointed out that thermal destruction of malignant cells needs energy to break chemical bonds (*E*_*R*_ in Figure 2). This part of the energy does not produce an increase of the temperature *T*. In principle, the total thermal energy absorbed in the tissue *U* (including the part used for chemical modifications) can be considered a thermal dose in analogy to the radiation dose *D*, which is the absorbed radiation energy per mass. Also in the case of radiation, not all the energies lead to chemical reactions since very weak interactions (especially between secondary electrons and atoms) are producing heat without molecular modifications. However, in clinical routine, temperature (for hyperthermia) and absorbed dose (for radiation therapy) are accessible to measurements and therefore used for dosimetric purposes.

The energy deposition in the cells leads to chemical reactions and therefore to a change of the (molecular or structural) configuration. This is incorporated in the general structure by a layer with configuration quantities. Referring to the concepts of statistical mechanics and thermodynamics, these configuration quantities are represented by variables of state. Unfortunately, the microscopic approach for calculating these quantities is, in contrast to ideal gases and crystals, very difficult or impossible. InSection 2.2, a proposal for a macroscopic approach to these state variables for heat and radiation will be made.

In the model framework shown in Figure 2, the configuration quantities or state variables influence the cellular system in two different ways. On the one hand, radiation and heat are damaging proteins with the result of a reduced repair capacity. On the other hand, radiation induced DNA damages are responsible for removing vital cells out of the mitotic cycle. In a more theoretic view, the variables of state are producing a “signal strength” which governs cell death and cellular repair. In the proposed concept, we distinguish clearly between variables of state in the sense of configuration quantities and information (here information about the biological impact upon the system). This information will be decoded at the level of the population, where the “signal strengths” directly influence the transformation of cells from vital to damaged cells.

The use of compartmental population models at the outcome level allows the inclusion of the mitotic cycle (mitotic cell population and cells in the G1-, S-, and G2-phase). The radiosensitivity of mitotic cells, cells in the G2-phase, and cells in the S-phase is different. Also subpopulations with different radiosensitivity are observed in malignant tumours. Therefore, to guide understanding, tumour response in patients and clinical outcome, population-based models are advantageous. However, in this paper a simplified model using one population of viable (tumour) cells is used to compare the survival to experimental data from defined cell lines.

### 2.2. Variables of State and Repair Probability

#### 2.2.1. Description of Radiation Induced Protein-Related Damages

The key idea of the Γ-LQ model [18] is the substitution of the absorbed dose by a biological dose equivalent Γ. In the framework of the Γ-LQ model, the dose equivalent Γ is assumed to be proportional to the average number of unrepaired sub-lethal entities per cell produced by irradiation. The discussion in [18] focused on DNA damage repair kinetics, since the dose equivalent was intended to describe DNA-related damages. The model described in this paper uses a different approach. The dose equivalent Γ is dedicated to describing protein-related, radiation induced damage. It is assumed that this dose equivalent increases linearly with the dose rate *R*. According to the Γ-LQ model, the following kinetic model is chosen for Γ [18]:

(1) $$\begin{array}{c} \frac{d\Gamma }{dt}=R-f\left(\Gamma \right). \end{array}$$

Here, *f*(Γ) is a function of Γ representing the kinetics of repair of protein-related damage. In principle, different types of repair kinetics may be taken into consideration. In the following, a simplistic approach using first-order kinetics is used: *f*(Γ) = *γ*Γ. The biological dose equivalent can be calibrated to the absorbed dose. By using this calibration, Γ must satisfy the following condition:

(2) $$\begin{array}{c} \underset{t\to \infty }{\lim}\left[\int_{-\infty }^{t}f\left(\Gamma \left(\tau \right)\cdot \right)d\tau \right]=\underset{t\to \infty }{\lim}\left[D\left(t\right)\right]=D_{\text{tot}}. \end{array}$$

For the biological dose equivalent, the unit of the absorbed dose Gray (Gy) can be used. The implicit assumption behind this definition is that no saturation of damages will be achieved by radiation. For very high doses (typically above 200 Gy), this is possibly not the case, since all cellular structures seem to be damaged in a severe manner and even membrane lipids might play an important role for interphase death at such high doses [27]. In the formalism described in the following section, Γ is used to describe only the impact on proteins involved in the repair of DNA-related lesions. Therefore, the concept is limited to DNA-related cell death mechanism (mitotic cell death, aspects of apoptotic cell death will be discussed later).

#### 2.2.2. Description of Heat-Induced Protein-Related Damages

In contrast to radiation, the energy deposition during heating of tissues/cells occurs in a distributed manner. All molecules absorb energy. By exceeding a temperature of 45–46°C (depending on tissue type), proteins will be heavily affected by heat. This leads to a different type of description of damages. Functional proteins can be converted by conformal changes or more generally by chemical reactions into nonfunctional forms. Johnson et al. [28] applied the Arrhenius law to enzymatic reactions and denaturation of enzyme protein. The activation energies of proteins in melanoma cell lines were determined from Arrhenius plots by Rofstad and Brufstad [29]. Above 43°C, an activation energy *E*_*a*_ of 700 kJ/mol was found. Below, the value for *E*_*a*_ varies between 1118 and 2190 kJ/mol. Based upon these findings, the following temperature depending, simplistic approach is chosen. The amount of functional repair proteins is described by a state variable *Υ*. If no thermal damage occurs and a maximal repair capacity is reached, this variable is set equal to 1:*Υ* = 0. If all repair protein molecules are damaged by heat and therefore are nonfunctional, the value should be *Υ* = 0. In this case, a variable Λ describing the amount of nonfunctional, damaged repair proteins is set to the value 1. The following system is assumed to describe the dynamics of thermal induction and repair (e.g., by Chaperones) of protein damages:

(3) $$\begin{array}{c} \frac{d\Upsilon }{dt}=-k_{1}\Upsilon +k_{2}\Lambda ,\\ \frac{d\Lambda }{dt}=k_{1}\Upsilon -k_{2}\Lambda ,\\ k_{1}=\kappa \cdot e^{-E_{a}/RT}, \end{array}$$

where *R* = 8.314 J · mol^−1^ K^−1^ and *T* is the temperature. The constants *k*_1_ and *k*_2_ are related to thermal degradation and repair of proteins. Here, first order kinetics is assumed. With these model assumptions, different types of repair proteins are not distinguished and only one value for activation energy is used. When going deeper into the processes of cellular repair, the formalism represented by (1) should be applied separately to different mechanisms of repair such as homologues recombination or nonhomologues end-joining repair. This point will be discussed later.

#### 2.2.3. Calculating Repair Probability Using Γ and Λ

According to the systemic structure in Figure 2, the variables of states Γ and Λ are configuration quantities describing radiation- and heat-induced damages (disorder). In a simplistic manner, they cover chemical and structural changes in the cell. The question arising is how these changes lead to a change of the cellular control processes. In Figure 2, the configuration quantities are distinguished from information, but in principle, the configuration quantities are encoding information about the state of the cellular system. This information converts in some way to signal strength for repair modification (increasing or reducing repair capacity). One possible approach is to define a probability of repair *P* which depends upon Γ and Λ : *P* = *P*(Γ, Λ). Moreover, we choose a very simplistic approach: the repair probability decreases monotonically with increasing values of Γ and Λ. Induced repair leading to low dose hypersensitivity is not considered. The following relations are used:

(4) $$\begin{array}{c} {\left[\frac{\partial P}{\partial \Gamma }\right]}_{\Lambda =\text{const}}=-\mu_{\Gamma }P,\\ {\left[\frac{\partial P}{\partial \Lambda }\right]}_{\Gamma =\text{const}}=-\mu_{\Lambda }P. \end{array}$$

This leads to the following functional dependence:

(5) $$\begin{array}{c} P\left(\Gamma \right)=P_{\Gamma }=e^{-\mu_{\Gamma }\Gamma },\\ P\left(\Lambda \right)=P_{\Lambda }=e^{-\mu_{\Lambda }\Lambda }. \end{array}$$

In the case of *P*_Γ_ and *P*_Λ_ being statistically independent, the total probability is given by

(6) $$\begin{array}{c} P=P_{\Gamma }P_{\Lambda }=e^{-(\mu_{\Gamma }\Gamma +\mu_{\Lambda }\Lambda )}. \end{array}$$

### 2.3. Multi-Hit-Repair Approach and Population Model

The transformation of the impact of radiation upon cell killing and the impact of radiation and heat upon cellular repair can be realised at the level of a population model. Vital cells (*N* = number of cells or population size) can be converted to damaged cells by radiation in the following way: the probability to hit the DNA is proportional to the number of cells in the population *N* and the dose rate *R*. The cell transformation rate is therefore given by $dN/dt=\dot{N}=-\alpha RN$ (using a radiosensitivity coefficient *α*). The cells affected by radiation will be removed from the mitotic cycle and converted to damaged cells (population size *L*_1_). It has to be pointed out here that the cells of population *L*_1_ are not considered lethally damaged cells and can be recovered by repair or converted to more damaged cells by a second (population size *L*_2_) and a third hit (population size *L*_3_). No criterion for lethality is applied here—lethality may be regarded as a result of a single hit or of several hits/damages, which will not be repaired. At this point, no exact definition for a “hit” is given (this will be discussed in Section 4). Applying this concept, the following system model describing a chain of populations can be derived:

(7) $$\begin{array}{c} \frac{dN}{dt}=-\alpha RN+r\left(L_{1}\right),\\ \frac{dL_{1}}{dt}=\alpha RN-\alpha RL_{1}-r\left(L_{1}\right)+r\left(L_{2}\right),\\ \frac{dL_{k}}{dt}=\alpha RL_{k-1}-\alpha RL_{k}-r\left(L_{k}\right)+r\left(L_{k+1}\right). \end{array}$$

The index *k* represents the number of hits. It is assumed that the probability of hits and subsequently the cell transformation rate are the same for all the populations. Moreover, the functions *r*(*L*_*k*_) introduced in (7) describing the rates of repair are assumed to be independent of the number of hits. The repair function *r*(*L*_*k*_) incorporates the repair probability equation (6) and, in the case of a first order process, may be written as *r*(*L*_*k*_) = *c*_*r*_e^−(*μ*_Γ_Γ+*μ*_Λ_Λ)^ · *L*_*k*_.

The model represented by (7) is incomplete since the elimination of cells after acquiring radiation induced damages is not considered. A prominent example of such an elimination process is the apoptosis. Apoptotic cell death can be regarded as a result of deactivated or not executed repair processes due to the activation of a separate elimination pathway. Such a process can be included in (7) by an additional elimination rate ${\dot{L}}_{k,e}$ which is assumed to be a linear function of the population size of damaged cells ${\dot{L}}_{k,e}=c_{e}L_{k}$:

(8) dNdt=−αRN+cre−(μΓΓ+μΛΛ)·L1,dL1dt=αRN−(αR+cre−(μΓΓ+μΛΛ)+ce)·L1+cre−(μΓΓ+μΛΛ)·L2,dLkdt=αRLk−1−(αR+cre−(μΓΓ+μΛΛ)+ce)·Lk+cre−(μΓΓ+μΛΛ)·Lk+1.

**Figure 3:**
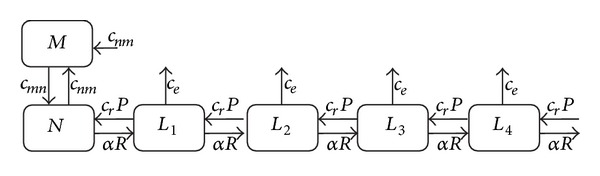
Illustration of the population model. The model flow chart includes a mitotic cell population as well (population size *M*). The flows (rates) between the populations can be found by multiplying the given constants by the corresponding population size (population where the arrow starts). *P* is the repair probability from (6).

These are in principle the core equations of the proposed Multi-Hit-Repair (MHR) model. The underlying structure of the model in (8) is illustrated in Figure 3. There, a mitotic cell population is included as well. For fast growing tumours *in vivo*, the inclusion of this population could be essential since a significant part of mitotic cells is present having a different radiosensitivity.

For practical reasons, the chain of populations has to be interrupted at a certain number of hits and the repair of the subsequent populations in the chain will be neglected. Including this cutoff, the model in (9) should be slightly modified. The last population in the chain is described by the following equation (where *k*_max⁡_ denotes the maximal value of *k* used for simulation):

(9) dLkdt=αRNk−1−(αR+cre−(μΓΓ+μΛΛ)+ce)·Lk+cre−(μΓΓ+μΛΛ)·Lk+1,dLkmax⁡dt=αRNkmax⁡−1−(αR+cre−(μΓΓ+μΛΛ)+ce)·Lkmax⁡.

The error resulting from the cutoff at *k*_max⁡_ will be investigated in Section 3.2.

## 3. Results

For Sections 3.1–3.3, only the radiation part of the model (8) is investigated (*P* is set to *P*_Γ_ and *P*_Λ_ = 1). In these sections, different radiobiological aspects are discussed. To give an overview, the used parameters are summarized in Table 1.

The differential equations (1) and (3) (see Section 3.4) and (8) are integrated numerically by a Runge-Kutta algorithm (4th order). The time steps were set to values between Δ*t* = 10^−3^ h and Δ*t* = 5 · 10^−5^ h.

If not otherwise indicated in the following sections, *k*_max⁡_ is set to a value of 6. In Section 3.2, the effect due to the cutoff at *k*_max⁡_ according to (9) is investigated for a typical radiobiological example.

If not stated otherwise, the parameters which we present for the different variants of investigated models have been obtained by evolutionary optimization procedures. For a set of parameters (*A*_1_,…, *A*_*n*_) with (*A*_1_,…, *A*_*n*_) either equal to (*α*, *c*_*r*_, *c*_*e*_, *γ*, *μ*_Γ_) or (*α*, *c*_*r*_, *c*_*e*_, *γ*, *μ*_Γ_, *κ*, *k*_2_, *μ*_Λ_), we apply the following procedure. First, we compute *N*(*A*_1_,…, *A*_*n*_, *t*_*R*_, *d*_*R*_, *t*_*H*_, *d*_*H*_, *t*). As before, *N* indicates the size of the population, *t*_*R*_ the time at which irradiation starts, *d*_*R*_ is the duration of the irradiation (determined by the dose), *t*_*H*_ represents the time at which the heating starts and *d*_*H*_ the respective duration (*t*_*H*_ and *d*_*H*_ are omitted in pure RT models). Durations and time intervals are chosen such that they correspond to experimental data. Differences between the logarithms of computed and measured values are squared and summed up; this sum constitutes the fitness function *f*(*A*_1_,…, *A*_*n*_) which is to be minimized. We employ a rather simple evolutionary procedure. Let (*A*_1_^*i*^,…, *A*_*n*_^*i*^) the parameters of the *i*th generation. We set (*A*_1_^*i*+1^,…, *A*_*n*_^*i*+1^) = ((1 + *ε*(*i*))*r*_1_^*i*^ *A*_1_^*i*^,…, (1 + *ε*(*i*))*r*_*n*_^*i*^ *A*_*n*_^*i*^) with *r*_*k*_^*i*^ a uniformly distributed random variable between −1 and 1. The parameter *ε*(*i*) is a small number which is reduced over the course of the evolution. We used 10^5^ steps, *ε*(*i*) was set to 0.02 for the first 66000 steps and then reduced to 0.01. If *f*(*A*_1_^*i*+1^,…, *A*_*n*_^*i*+1^) ≤ *f*(*A*_1_^*i*^,…, *A*_*n*_^*i*^), (*A*_1_^*i*+1^,…, *A*_*n*_^*i*+1^) remained unchanged; otherwise it was set back to (*A*_1_^*i*^,…, *A*_*n*_^*i*^). The method is basically a simple gradient search, which we have chosen because the fitness landscape turned out to be smooth, though flat. This flatness means that parameter variations most often have only little impact upon the outcome and therefore the results of our optimizations have to be interpreted with some caution. Our choices fit well, but different sets of parameters fit almost as well. In order to cope with the possibility of local optima, we performed the optimization several times with different initial values. We observed convergence into the same optimum and therefore concluded a simple gradient search to be appropriate for the models we investigated. However, further improvements, for example, the addition of additional mechanisms, may require more sophisticated optimization methods. A method suitable for chemical systems (i.e., systems with an in general smooth fitness landscape) with a number of continuous parameters comparable to the models presented in this work is presented in Forlin et al. [30]. Not all chemical systems have a smooth fitness landscape and this holds even more for biological systems such as eukaryotic cells with versatile functionality. A method applicable for categorical as well as continuous systems and nonsmooth fitness landscapes is discussed in Ferrari et al. [31]. A general framework, ParamILS, for parameter tuning (or as Hutter et al. [32] prefer to call it, algorithm configuration) is described in [32]. ParamILS is suitable for numerical, ordinal, and categorical parameters. The ability to deal with categorical parameters is of specific value when different states of cells are not anymore connected in a sequential manner, but, for example, cell differentiation is part of the model.

### 3.1. Apoptotic Cell Death versus Nonapoptotic Cell Death

**Figure 4:**
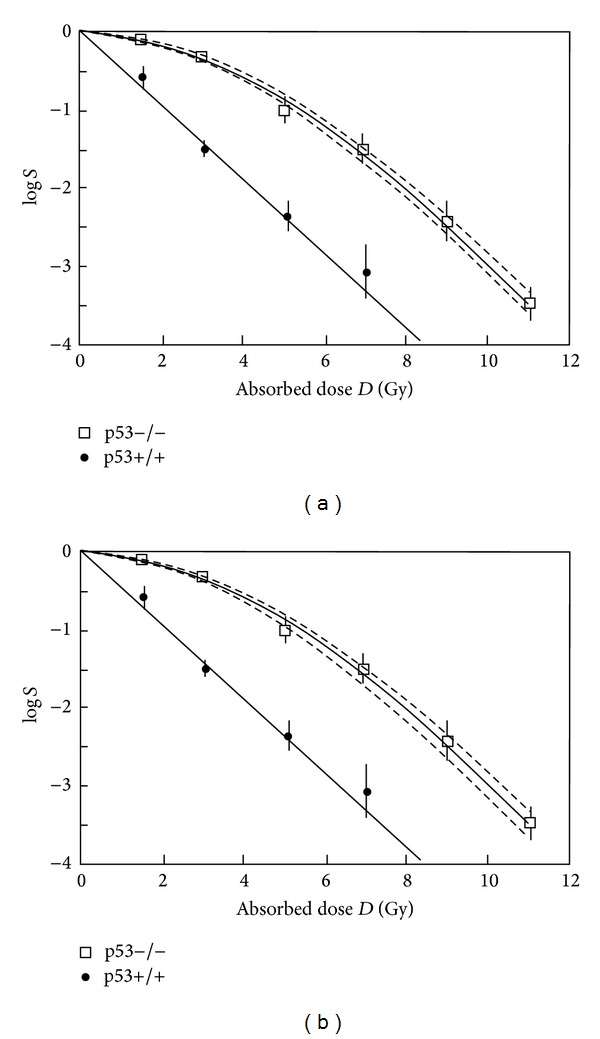
Fit of cell survival in the case of apoptotic (p53+/+) and nonapoptotic (p53−/−) cell death. The solid straight line is given by log⁡⁡*S* = −(1.1 · log⁡*e*) · *D*. The dashed lines indicate the standard deviation calculated for varying repair parameters, (a) variation of *c*_*r*_ between 84 and 120 h^−1^; (b) variation of *μ*_Γ_ in the range of 0.45–0.55 Gy^−1^.

Apoptotic cell death can be characterised by a linear function with a steep slope in the logarithmic cell survival curve. In the framework of the MHR model in (8) and Figure 3, this behaviour can be interpreted as an efficient elimination process (described by the elimination constant *c*_*e*_), which prevents a repair of severe or critical cellular lesions. In this case, the equation describing the vital cell population *N* simplifies to *dN*/*dt* = −*αR* *N* and therefore ln⁡*S* = −*α*D. In principle, the case with apoptotic cell death can be considered as a baseline cell killing according to Figure 1(b). Data from apoptotic cell killing can be used to determine the baseline radiobiological constant *α*. In Figure 4, a fit of data from Hardenbergh et al. [8] is shown. The p53 wild type (nondeficient, radiation sensitive) murine fibroblast exhibits the typical linear relationship with a steep slope. The radiosensitivity coefficient *α* was determined by Harrigan et al. (*α* = 1.1 Gy^−1^ for the natural logarithm of *S* : ln⁡*S* = −*αD*). For the fit of the data by the model in Table 1, the same value is used. The p53-deficient, radioresistent fibroblasts are characterized by a linear-quadratic behaviour in the logarithmic survival plot. The radiosensitivity coefficients were determined by Harrigan et al. to *α* = 0.13 Gy^−1^ and *β* = 0.054 Gy^−2^ (for ln⁡*S* = −(*α*D + *β*D^2^). For the data fit using the MHR model, the baseline value for *α*( = 1.1 Gy^−1^) is used. A good fit can be achieved with *c*_*r*_ = 100 h^−1^, *c*_*e*_ = 10 h^−1^, *μ*_Γ_ = 0.5 Gy^−1^, and *γ* = 1.45 h^−1^ at a dose rate of 60 Gy/h. To test the sensitivity of the MHR model to variations of the repair constants, *c*_*r*_ and *μ*_Γ_ are varied and the resulting standard deviations are given (dashed lines). To keep the standard deviation within the error limits of the experimental data, c_r_ can be varied by ±16% and *μ*_Γ_ can be varied in the range of ±10%, respectively.

### 3.2. Cutoff of Subpopulation with Damages

**Figure 5:**
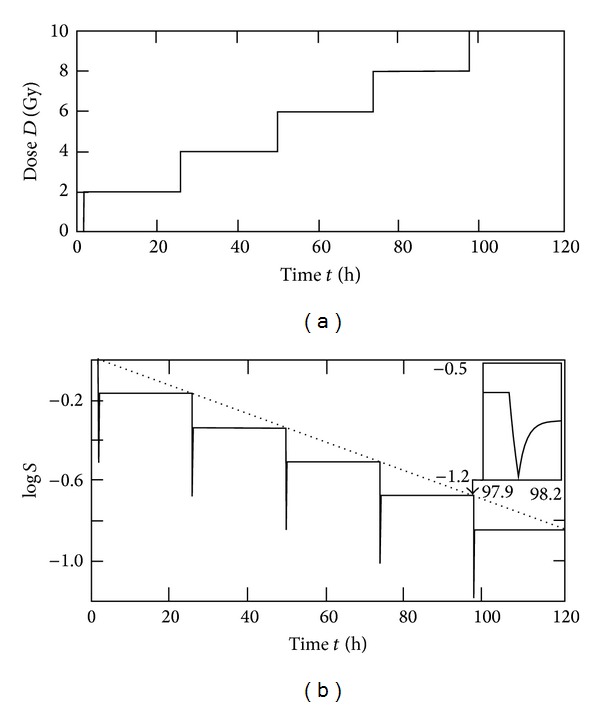
Fractionated radiotherapy course used for cutoff evaluation: (a) For every dose step (fraction of 2 Gy), a constant reduction of log⁡⁡*S* results. Therefore, the (upper) envelope in (b) is characterized by a straight line (dotted line in the lower figure). This corresponds with the concept of Oliver [7] and the radiobiological models of Curtis [15] and Scheidegger [18] and represents the case of complete repair.

For simulations, the chain of populations in the MHR model has to be cutoff at certain *k*_max⁡_ (cf.  Section 2.3, (9)). The related effect can be investigated for typical examples by comparing the resulting survival. For the evaluation, the parameters from Section 3.1 were used (nonapoptotic cell killing). For testing the influence of the dose rate *R*, the values of *R* were varied in the range of 14–240 Gy/h (typical range for conventional linear accelerators used in radiotherapy). A common radiotherapy course consists of several fractions of 1.5–2.5 Gy with spacing between the fractions of at least one day. Therefore, the cutoff effect was evaluated for 5 fractions of 2 Gy (at a dose rate of 60 Gy/h). The resulting logarithmic survival as function of time is shown in Figure 5. In agreement with the model of Curtis [15] and the Γ-LQ model [18], the envelope of the log⁡⁡*S*-graph is given by a straight line (upper envelope as dotted line in Figure 5).

To evaluate the cutoff effect also for different doses per fraction, fraction sizes of 8 Gy at a dose rate of 240 Gy/h were investigated.

**Figure 6:**
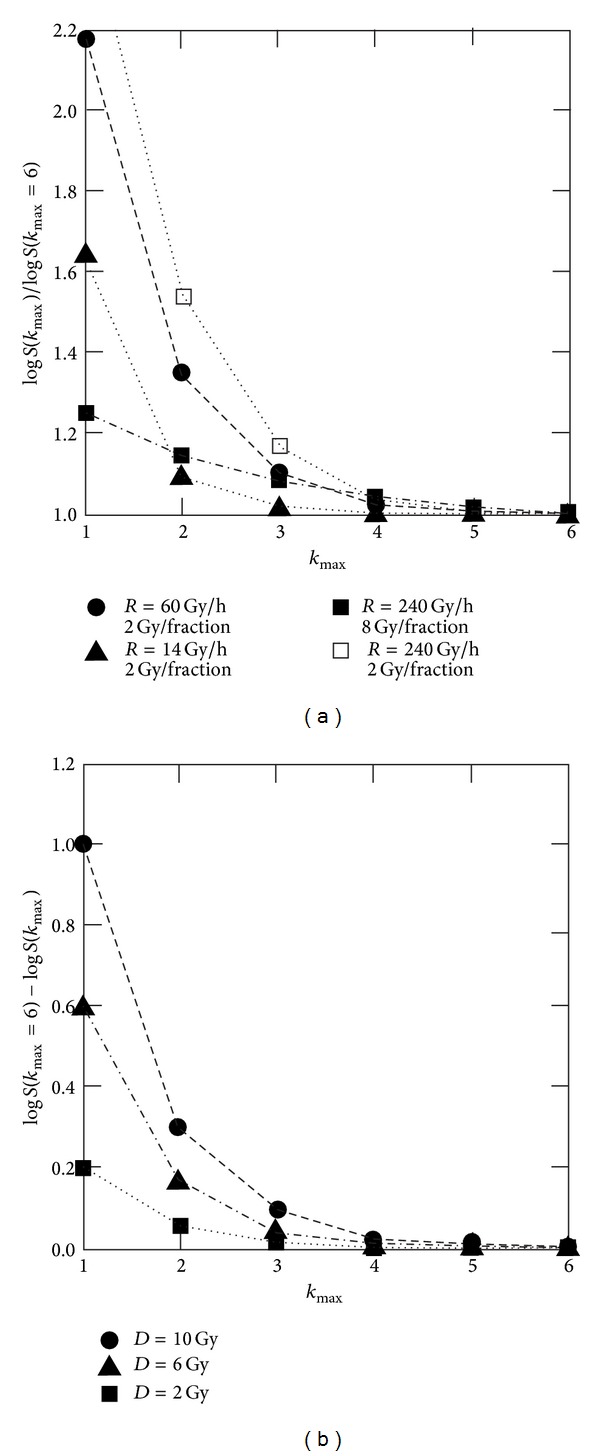
Effect of cutoff of the population chain at different dose rates *R*. In the left diagram, factors between log⁡⁡*S*-values for a specific *k*_max⁡_ and log*S*-values *k*_max⁡_ = 6 are shown. These values exhibit a nonlinear dose rate dependence but are nearly independent of the cumulative dose for a specific fractionation scheme (2 Gy fractions according to Figure 5). The situation becomes different for larger doses per fraction (example in the Figure 8 Gy fractions). Due to the high dose rate and the low *γ*-value (1.45 h^−1^), the dose equivalent Γ does not reach a steady state and rises up to approximately 8 Gy (7.83 Gy). This leads to a higher repair rate and therefore to a slightly higher influence of the populations with *k* < 1. In the right diagram, the differences of the log⁡⁡*S*-values are given. This quantity is dependent of the cumulative dose (in this figure applied in fractions of 2 Gy) and can be approximated by an exponential function (for the discrete values of *k*_max⁡_).

In Figure 6, the cutoff effect is illustrated by the coefficient log⁡⁡*S*(*k*_max⁡_)/log⁡*S*(*k*_max⁡_ = 6) and the difference Δlog⁡*S* = log⁡⁡*S*(*k*_max⁡_ = 6) − log⁡*S*(*k*_max⁡_) = log⁡(*S*(*k* = 6)/*S*(*k*_max⁡_)). In the case of the coefficient (Figure 6(a)), the influence of different doses per fractions or different cumulative doses is small. The maximum variation was found for *k*_max⁡_ = 1 and varying doses between 8 and 40 Gy at a dose rate of 240 Gy/h: average ± standard deviation of the factor is log⁡⁡*S*(*k*_max⁡_ = 1)/log⁡⁡*S*(*k*_max⁡_ = 6) = 1.2516 ± 0.0045. This quantity seems to be more or less independent of the dose in the tested range (2–40 Gy). The effect of the dose can be shown by the difference Δlog⁡ *S* (Figure 6(b)). The relationship between ΔlogS and the (discrete) values of *k*_max⁡_ can be approximated by an exponential function (in the case of *D* = 10 Gy Δlog⁡⁡*S* = 1.001 · *e* ^−3.72·*k*_max⁡_^ for all data points and Δlog⁡⁡*S* = 1.001 · *e* ^−3.37·*k*_max⁡_^ for *k*_max⁡_ ∈ {1,2}).

For all investigated cases, the cutoff effect becomes small for *k*_max⁡_ > 4. The reason for this behaviour lies in the structure of the MHR model. The probability for recovery of cells (transformation back to the population *N*) with more than 4 impacts (radiation induced DNA lesions) can be neglected compared to the elimination rate *c*_*e*_ *L*_*k*_.

### 3.3. Dose Rate Dependence

**Figure 7:**
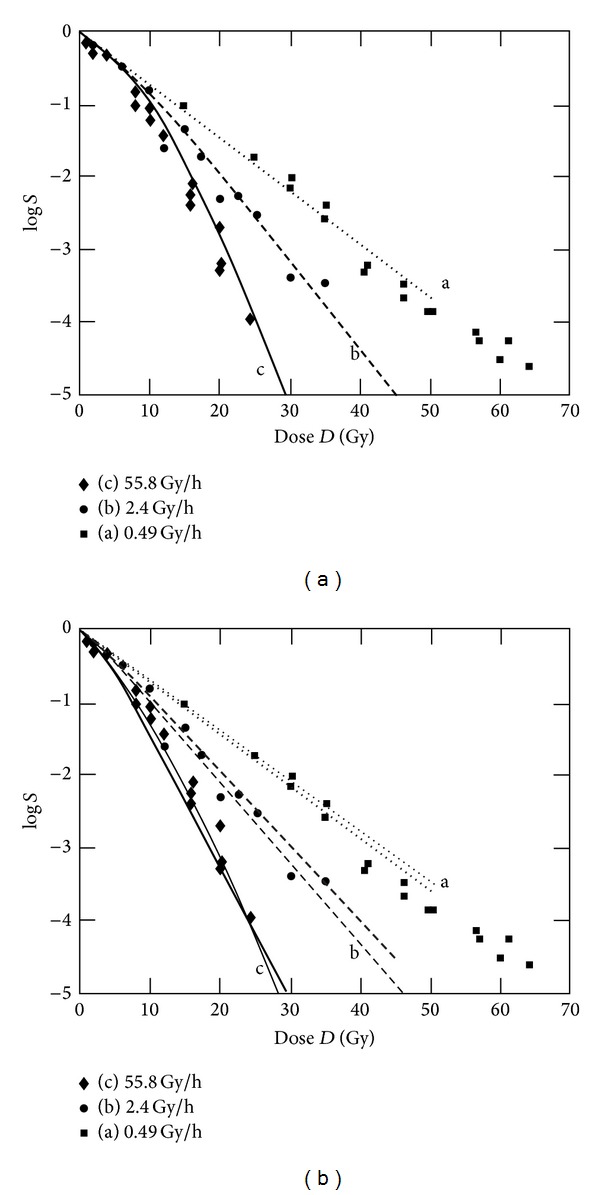
Fit of experimental data form Wells and Bedford [5]. C3H10T1/2 cells with the LQ parameters *α* = 0.1366 Gy^−1^ and *β* = 0.02 Gy^−2^, irradiated at different dose rates. The parameters used for fitting are given in Table 2. (a) shows a fit which corresponds to the fit of Curtis [15] using the LPL model, (b) shows two, not optimized fits with higher values for *c*_*r*_ and *c*_*e*_.

It can be expected that a model using five free parameters is able to fit linear-quadratic curves since a two-parameter law is doing so as well. Fitting of linear-quadratic-linear data obtained for high doses per fraction is more difficult [4]. An important test of a radiobiological model is given by its dynamic behaviour at different dose rates. We carried out this test by fitting experimental data from Wells and Bedford [5]. The results are shown in Figure 7.

A good fit can be obtained with parameter values given in Table 2 (values for Figure 7(a)). Compared to the p53-deficient murine fibroblasts in Section 3.1, the C3H10T1/2 cells have a smaller value for the LQ parameter *β* (0.02 Gy^−2^ instead of 0.054 Gy^−2^) and a slightly higher value for *α*. This in principle leads to a less pronounced shoulder compared to larger *β* values. In comparison with the parameter values for the MHR model, the values of *c*_*r*_ and *c*_*e*_ in Figure 7(a) differ significantly from those used for the murine fibroblasts (*c*_*r*_: 0.51 h^−1^ instead of 100 h^−1^ and *c*_*e*_: 0.14 h^−1^ instead of 10 h^−1^). For testing parameter values for *c*_*r*_ and *c*_*e*_ which are closer to those in Sections 3.1 and 3.2 and not optimized fits with higher values are carried out as well. The results for two selected cases are shown in Figure 7(b). The corresponding parameter values are given in Table 2. In this set of parameters, the values for *c*_*r*_, *μ*_Γ_, and *γ* are identical to the parameter values used in Section 3.1. Depending on the weighing of the data points and allowed parameter range, different sets of parameter values can be found to achieve a (more or less good) fit of the dose rate dependence.

**Figure 8:**
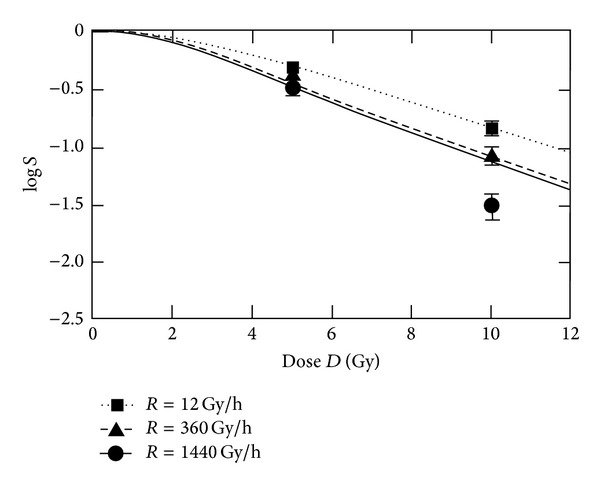
Clonogenic survival of T98G glioblastoma cells at different dose rates. The parameter values for fitting are *α* = 0.27 Gy^−1^, *γ* = 1.45 h^−1^, *c*_*r*_ = 90 h^−1^, *c*_*e*_ = 19 h^−1^, and *μ*_Γ_ = 0.8 Gy^−1^.

A good radiobiological model should fit the dose rate dependencies over a certain range. But for simplistic models, a limitation due to the different processes involved in the cellular response onto radiation can be expected. In particular for very high dose rates or high doses per pulse, changes in the radiobiochemical cascade cannot be excluded. Today, new linear accelerators for clinical use with flattening filter free (FFF) beams are available. To investigate possible biological effects, Lohse et al. [33] treated glioblastoma cell lines with doses of 5 and 10 Gy. The highest dose rate values are clearly above the range given in Table 1 (up to 1440 Gy/h). It has to be pointed out that a pulsed beam with high doses per pulse was used. In this paper, the data obtained from these experiments are used to explore the high dose rate limit of the MHR model. In Figure 8, a fit of the logarithmic survival of T98G glioblastoma cells irradiated at different dose rates is shown.

At very high doses, the log*S* curves in Figure 8 are exhibiting a linear-quadratic-linear shape. For dose rates above 240 Gy/h, the values for log*S* are changing only in a small range (−1.113 at 240 Gy/h; −1.129 at 360 Gy/h; −1.158 at 1440 Gy/h; −1.168 at 3000 Gy/h). The point at 10 Gy and 1440 Gy/h clearly cannot be fitted by this parameter set. With changed parameters, a fit of the 1440 Gy/h-data is possible for the price of a poor fit of the points at 12 Gy/h. No parameter set enabling a fit of all data points was found by the applied optimisation algorithm.

### 3.4. Inclusion of Hyperthermia

**Figure 9:**
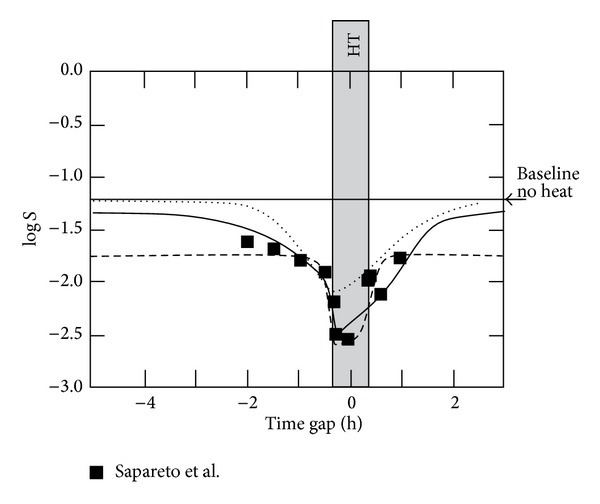
Fit of experimental data (redrawn) from Sapareto et al. [13]. Chinese hamster cells were irradiated with 5 Gy prior (negative time gap) or after heat (positive time gap). Heat (HT) is applied during 40 min (±20 min of point 0 on the time gap axis). Temperature *T* during heating was 42.5°C. Heat specific parameter values are given in Table 3. Radiation specific parameter values: solid line (optimized) *α* = 1.89 Gy^−1^, *c*_*r*_ = 191.7 h^−1^, *c*_*e*_ = 0.97 h^−1^, *μ*_Γ_ = 0.96 Gy^−1^, *γ* = 6.77 · 10^−3^ Gy^−1^; dotted line (not optimized) *α* = 1.1 Gy^−1^, *c*_*r*_ = 6.1 h^−1^, *c*_*e*_ = 2 h^−1^, *μ*_Γ_ = 0.5 Gy^−1^, *γ* = 1.45 Gy^−1^; dashed line (optimized without baseline points) *α* = 1.18 Gy^−1^, *c*_*r*_ = 6.42 h^−1^, *c*_*e*_ = 8.92 h^−1^, *μ*_Γ_ = 0.096 Gy^−1^, *γ* = 0.699 Gy^−1^.

The synergistic effect of heat and radiation depends upon the time gap between application of hyperthermia and radiation [13]. A model for the synergistic effect should cover this dynamic aspect. To test the dynamic behaviour of the extended MHR model, the experimental data from Sapareto et al. [13] are used. With respect to Table 1, the inclusion of the effect of heat in the MHR model requires additional parameters, which are summarized in Table 3. Selected results of different calculations (with and without using evolutionary optimization) are shown in Figure 9.

The parameter optimisation using only the data points given by Sapareto et al. leads to a shifted baseline (dashed line, Figure 9). It can be assumed that with a sufficient large time gap, the cell survival would be the same as without applying heat (baseline at log⁡⁡*S* = −1.21). This assumption is included in the fit represented by the solid line in Figure 9. Two additional, hypothetical baseline points (with double weight) at a positive and negative time gap of 7 h are used for fitting. The resulting graph covers more or less the experimental data points. Comparing the corresponding parameter values found by the optimisation algorithm with those ones in Sections 3.1–3.3, clearly different values for *γ* (very small), *c*_*r*_, and *c*_*e*_ are resulting. A solution (not optimized fit) which is closer to the parameter values of Sections 3.1–3.3 is shown as dotted line in Figure 9. This graph starts at the baseline and recovers the baseline when radiation is applied after heat with a time gap larger than 2 h. Parameter sets similar to those used in the previous sections generally lead to a smaller effect (shifted baseline and covering the data points at log⁡⁡*S* = −2.55 or baseline at log⁡⁡*S* = −1.21 and maximal cell killing at log⁡⁡*S* = −2.0).

## 4. Discussion and Conclusions

Our hypothesis—that a model reflecting dynamic interaction of DNA and repair proteins (such as the MHR model) is able to describe aspects of the impact of radiation such linear-quadratic (-linear) behaviour and dose rate dependence of survival—can be confirmed with some restrictions. The MHR model is exhibiting linear-quadratic and linear-quadratic-linear behaviour as observed in radiobiological experiments. It catches in an intuitive way the behaviour of apoptotic and nonapoptotic (radiation induced) cell death. Also, fractionation of radiation as is usual for standard radiation therapy is covered correctly.

Regarding the chain of populations which is used in the model, at least populations up to 4-5 hits (radiation induced, potentially lethal DNA damaged) should be included. No specifications about radiation induced damages (“hits”) are made. Regarding the fact that a dose of 1 Gy (X-rays) is producing ~25 double strand breaks, a “hit” cannot be equivalent to a double strand break in general. Since the probability of a second double strand break in the proximity of a previously induced double strand break is proportional to *D* ^2^, a hit also cannot be interpreted as the formation of a damage consisting of two double strand breaks with a certain special correlation. Possible interpretations of the term “hit” may take into account that possibly not all locations on a chromosome have the same sensitivity for formation of severe chromosomal damages due to double strand breaks. In addition, the different (protein- and DNA-related) parts of the MHR model could refer to the different pathways of repair. We cannot exclude the possibility that a certain (very fast) repair process (with a small sensitivity to protein damages) is included as well in the *α* coefficient. Otherwise, one would expect a similar *α* value for all cell lines which should reflect a cell killing according to the approximately 25 double strand breaks per Gy. Assuming Poisson statistics, the surviving fraction is given by the number of unrepaired lesions *n*: *S* = *e* ^−*n*^ = *e* ^−*αD*^. Without any repair process, the baseline cell killing would be described by *α* = 25 Gy^−1^. This is clearly higher than the values used for fitting in Section 3.1, where the baseline was determined from the apoptotic cell line.

The probability of induction of DNA-related lesions is assumed to be constant (represented by the parameter *α*). This represents in principle the case of independent events. We cannot exclude the possibility that previous damage events affect subsequent hits and *α* varies with the population order *k*. If the induction of different types of potentially lethal DNA-related lesions and their probability to be repaired is different, the model structure (population chain) will transform to a tree-structure consisting of branches of damaged populations with a specific *α*.

The (observed) dose rate dependence of cellular survival can be fitted at least in a limited range. The comparison of the experimental data of Wells and Bedford [5] exhibits a problematic aspect of fitting biological data by using the MHR model. Different weighting of data points leads to different sets of parameter values. Especially the values for *c*_*e*_ and *c*_*r*_ show a wide spread. The uncertainty of biological data seems to have a strong impact on the sharpness of the parameter values.

The dose and dose rate limitations may be cell line dependent. Especially the observation of an increased cell killing effect of glioblastoma cells at very high dose rates and high dose values per fraction (10 Gy) seems not to be covered correctly by the model. Reasons for this could be related to the triggering of different chemical reactions in the radio-biochemical cascade at very high doses per pulse, radiation induced, severe damage of mitochondria with subsequent energy depletion or destruction of other cellular structures. Regarding the radio-biochemical cascade, changes of instantaneous levels of radicals may not be affected since radical formation occurs within 10^−14^ s and recombination of H and OH radicals (and production of H_2_ and H_2_O_2_) is starting at 10^−12^ s [21]. Therefore, potential dose rate dependent modifications of the radio-biochemical chain are expected in subsequent steps. It has to be pointed out here that the additional cell killing effect at very high doses per pulse was observed for a few cell lines (T98G and U87MG glioblastoma cell lines which are very radio resistant) and FFF beams [33]. Some experimental absence of dose rate dependence at ultrahigh dose rates is found by different authors [34, 35]. In a review article, Ling et al. [20] concluded that the dose rate effect in external radiotherapy is governed by the beam-on time, not by the average linear accelerator (linac) dose rate, nor by the instantaneous dose rate within individual linac pulses (even for FFF machines). This is an important point especially for intensity modulated radiotherapy (IMRT) where during beam-on time not the whole target volume is irradiated due to time dependent beam collimation. In principle, the target dose in IMRT or in high precision radiotherapy [36] is applied in a prolonged way compared to non-IMRT treatments. In this view, the results of the MHR model are in agreement with these findings. Variations in the dose rate above 240 Gy/h have only a small impact. Time gaps during irradiation due to changing beam collimations have a similar impact like lowering the dose rate to values where the model shows stronger dose rate dependence.

**Figure 10:**
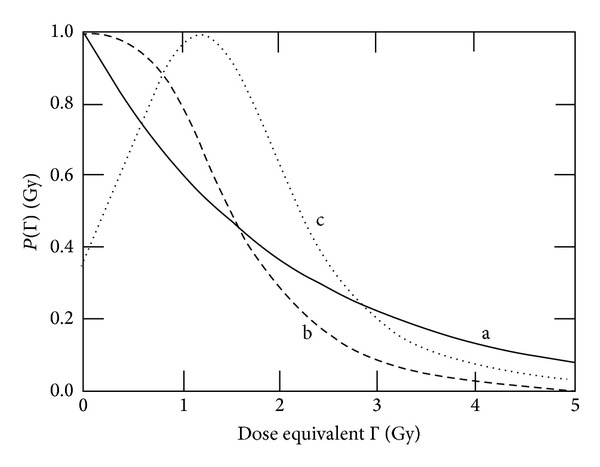
Different approaches for describing the dose equivalent dependence of the repair probability. (a) Exponential function with *μ*_Γ_ = 0.5 Gy^−1^ as used in Sections 3.2−3.4, (b) sigmoidal function, and (c) possible function in the case of low dose hypersensitivity.

The assumption of a monotonically decreasing repair probability (4) and (5) is questionable. To model low dose hypersensitivity, a radiation induced repair process should be considered. Such a process can be covered by a Gaussian function using a characteristic dose Γ_*c*_ [23]: *e* ^−*ξ*(Γ−Γ_*c*_)^2^^. This leads to a modified model and a different (8): *dL*_*k*_/*dt* = *αR* *L*_*k*−1_ − (*αR* + *c*_*r*_ · *e* ^−*ξ*(Γ−Γ_*c*_)^2^^ + *c*_*e*_) · *L*_*k*_ + *c*_*r*_ · *e* ^−*ξ*(Γ−Γ_*c*_)^2^^ · *L*_*k*+1_. Moreover, sigmoidal courses of *P*(Γ) should be considered (Figure 10). A rationale for such an approach may be the assumption of a certain stability of repair protein at low doses. Also a combination of the different patterns of dose-equivalent dependencies could be taken into consideration, since radiation affects all proteins. The initial induction of protein-related damage may be unspecific when regarding direct ionisation and possibly more specific when damages occur via radical formation (indirect pathway). The impact upon the repair capacity (covered by the repair probability) is dependent upon the sensitivity of the different repair pathways regarding radiation induced damage. As a consequence, a more or less unspecific initial damage process would transform into a protein (and pathway) specific response.

The MHR model is a cell population based approach and can therefore be extended to a tumour model with subpopulations characterized by different radiosensitivity. To model effects of cell cycle synchronisation, the inclusion of mitotic cells is important as well. Such extensions may possibly be very important to understand the tumour dynamics *in vivo.* Varying radiosensitivity of different groups of cells may override small differences, for example, due to dose rate dependence above 240 Gy/h. When evaluating tumour volumes of irradiated tumours *in vivo*, the mixture of tumour cells with different radiosensitivity could be essential. In contrast, the overall survival may be governed by the subpopulation with the lowest radiosensitivity.

The verification of the model including the synergistic effect of heat and radiation is difficult. By fitting the experimental data from Sapareto et al. [13], unexpected parameter values were found (compared to those ones used for radiation only). Fits with parameter values close to the Sections 3.1–3.3 are able to cover the experimentally observed time gap dependence, but only with a shift of the maximum cell killing or the baseline in the order of ΔlogS = 0.5. One reason could be an additional, less time gap dependent (slower) process which is not covered by the model. Another explanation of the shift of the baseline is that this shift is an artefact of the optimisation procedure, which only considers the measured data points, meaning mostly points off the baseline. In principle, this weakness of the evolutionary procedure is easily resolved by introducing “artificial” data points on the baseline for very large time gaps. However, such a procedure should be properly justified and corroborated by some experiments. A better estimation of the parameter values and validation of the model is only possible by using better experimental data. Such data should be generated with a clearly characterized cell line and should not only include time gap dependence but also dose rate dependence (with and without radiation). In addition, time resolved data about DNA- and protein-related repair would help to validate the proposed approach.

From a clinical perspective, future research should refer the problem of thermal dose concept based on variables of state. Recently in the clinical routine applied thermal dose concepts such as the thermal isoeffect dose method (cumulative equivalent minutes (CEM) concept [37]) are completely misreading aspects of dynamic interplay between radiation and heat or varying time gaps between application of heat and radiation.

A remaining question is how to cover complex interaction of damage induction and repair by variables of state. Specific experiments dedicated to this question and delivering dynamic (time resolved) data about protein damages and repair activity would be helpful to refine the concept of state variables. The hypothetic impact of possible differences between the radiation induced response of different cell lines found by such experiments could be evaluated by the (modified) MHR model. In this context, the MHR model offers a work bench for testing concepts and ideas about the control of cellular repair.

## Figures and Tables

**Table 1:** Parameters in use for the radiobiological investigations of Sections 3.1–3.3. The typical range in column 3 represents the range used for fitting radiobiological data.

Parameter	Related equation	Typical range
*α*	$\frac{dN}{dt}=-\alpha RN+c_{r}e^{-(\mu_{\Gamma }\Gamma )}\cdot L_{1}$ ⋮ $\frac{dL_{k}}{dt}=\alpha RL_{k-1}-\left(\alpha R+c_{r}e^{-(\mu_{\Gamma }\Gamma )}+c_{e}\right)\cdot L_{k}+c_{r}e^{-(\mu_{\Gamma }\Gamma )}\cdot L_{k+1}$	0.5–2 Gy^−1^
*c*_*r*_	Same as for *α*	4–100 h^−1^
*c*_*e*_	Same as for *α*	1–60 h^−1^
*μ*_Γ_	Same as for *α*	0.2–1.0 Gy^−1^
*γ*	$\frac{d\Gamma }{dt}=R-\gamma \Gamma$	1–10 h^−1^
*R*	Same as for *α* and *γ*	0.49–240 Gy/h

**Table 2:** Parameters used for fitting dose rate dependent data from Wells and Bedford [5].

Parameter	Figure 7(a)	Figure 7(b) thick (thin) lines
*α*	0.79 Gy^−1^	0.42 (0.7) Gy^−1^
*c*_*r*_	0.51 h^−1^	100 (4) h^−1^
*c*_*e*_	0.14 h^−1^	55 (1) h^−1^
*μ*_Γ_	0.036 h^−1^	0.5 (0.5) Gy^−1^
*γ*	9.23 h^−1^	1.45 (1.45) h^−1^

**Table 3:** Additional parameters to model synergistic effect of heat and radiation.

Parameter	Related equation, remarks	Value used for Figure 9 solid (dotted; dashed) lines
*κ*	*k*_1_ = *κ* · *e* ^−*E*_*a*_/*RT*^ *κ* is specific for *T* < 43°C, *T* > 43°C is not used in Figure 9	$\frac{a\cdot 10^{-3}\;{\text{h}}^{-1}}{e^{-(E_{a}/(R\cdot (38+273.16)K))}}$ with *a* = 0.56 (1; 0.89)
*E*_*a*_	Same as for *κ* (3)	1528 kJ for *T* < 43°C [23]
*k*_2_	$\frac{d\Upsilon }{dt}=-k_{1}\Upsilon +k_{2}\Lambda$; $\frac{d\Lambda }{dt}=k_{1}\Upsilon -k_{2}\Lambda$	2.76 (2; 11.17) h^−1^
*μ*_Λ_	(8)	29.19 (20; 31.26)
